# Child safety from the perspective of essential needs

**DOI:** 10.1590/0104-1169.3651.2458

**Published:** 2014

**Authors:** Débora Falleiros de Mello, Nayara Cristina Pereira Henrique, Letícia Pancieri, Maria de La Ó Ramallo Veríssimo, Vera Lúcia Pamplona Tonete, Mary Malone

**Affiliations:** 1 PhD, Associate Professor, Escola de Enfermagem de Ribeirão Preto, Universidade de São Paulo, WHO Collaborating Centre for Nursing Research Development, Ribeirão Preto, SP, Brazil; 2 RN; 3 PhD, Professor, Escola de Enfermagem, Universidade de São Paulo, São Paulo, SP, Brazil; 4 PhD, Professor, Departamento de Enfermagem, Faculdade de Medicina de Botucatu, Universidade Estadual Paulista "Júlio de Mesquita Filho", Botucatu, SP, Brazil; 5 PhD, Professor, Florence Nightingale School of Nursing and Midwifery, King's College, London, England

**Keywords:** Child, Health Promotion, Nursing

## Abstract

**OBJECTIVE:**

to characterize the maternal care for children under one year of age with a view to child health promotion at home.

**METHOD:**

exploratory study with qualitative data analysis, thematic mode, based on the conceptual framework of the essential needs of children, based on interviews recorded with 16 mothers.

**RESULTS:**

the analysis of the maternal narratives showed elements that facilitate the promotion of child safety: presence and involvement of the parents, constant surveillance for physical and emotional protection, experiences to stimulate child development, support networks for childcare at home; and inhibiting elements of child safety: limited perception of characteristics of child development and of children's singularities, overprotection and difficulties to set limits.

**CONCLUSION:**

the study enhances the understanding of home care in child health promotion, directing professional actions to guarantee ongoing nurturing relationships, protection, respect for individual differences, experiences appropriate to child development, limit setting and construction of stable and supportive social networks. In addition, the relevance of considering the maternal perspective in child health care is considered, as a strategy to apprehend aspects related to the attendance of the growth and development needs, particularly for child health promotion at home.

## Introduction

The safety and protection of children and their implications for health are current themes in research^(^ 1 ^-^ 5 ^)^, and the study of child safety has also stood out, given the range of morbidity and mortality levels due to external causes in childhood^(^ 3 ^)^, the importance of safe practices at home^(^ 4 ^)^, the concern with the quality of the environment the child lives on and the impact on his/her development^(^ 5 ^)^. Thus, the promotion of child health in the domestic sphere is extremely relevant and represents a challenge for health professionals^(^ 3 ^)^, especially in the context of primary health care. 

The actions focused on child health should be associated not only with survival, but mainly with the person's holistic development. Childcare, particularly in the first years of life, is fundamental for them to grow and develop healthily, be physically healthy, emotionally safe and respected as social subjects^(^ 6 ^)^. In the growth and development process, it is essential to acknowledge the double importance of physical and emotional health^(^ 1 ^)^. 

In primary health care, childcare, with regard to health promotion, including child safety, aims to guarantee child growth and development monitoring and surveillance, with care integrality and longitudinality, and mainly considering maternal and family values^(^ 7 ^)^. The knowledge and reflections about the subjects' needs, which often go by unnoticed or are reduced to demands modulated by health service supplies^(^ 8 ^)^, are vital for the understanding and feasibility of the care process. Therefore, it is fundamental for the professionals to appropriately acknowledge the individuals' needs and offer resources to attend to these needs.

Aspects of child care and safety, in view of the children's needs, have been hardly disseminated, particularly of children living in contexts of socioeconomic deprival and cultural diversity, with high levels of unattended health needs^(^ 1 ^,^ 9 ^)^ and injury risks^(^ 4 ^)^. Admitting the vital role of caregivers in the management of injury risks for small children^(^ 4 ^)^, it is relevant to investigate what care is performed to attend to their health and safety needs in those contexts, given their potential for unsafe environments. In that sense, the objective in this investigation was to characterize the maternal care for children of less than one year of age with a view to child health promotion at home, under precarious social conditions.

## Method

Exploratory study with qualitative data analysis, based on the conceptual framework of the essential needs of the children^(^ 10 ^)^.

The conceptual framework of the essential needs of the children in the health promotion framework involves the apprehension of the needs into: Need for ongoing nurturing relationships; need for physical protection, safety and regulation; need for experiences tailored to individual differences; Need for experiences appropriate to child development; Need for limit setting, structure and expectations; Need for stable and supportive communities and cultural continuity^(^ 10 ^)^.

The need for ongoing nurturing relationships refers to the presence of the child's caregiver and the form of constant interaction with the child, through physical care and affective interactions. The need for physical protection and safety aims to guarantee favorable conditions to maintain the child's physical and physiological integrity, involving food, hygiene, sleep, shelter, movements, growth and development monitoring, support for healthy habits and protection against infections and accidents, as well as regulations based on laws and other measures that protect the child against physical, social and environmental damage. The need for experiences tailored to individual differences is related to the supply of care particular to each child, excluding any form of standardized expectation. The need for experiences appropriate to child development involves actions to stimulate and add new interactions to an evolutionary process of each child's individual demand, allowing the children to gain self-confidence and feel accepted, cared for and loved. The need for limit setting, structure and expectations refers to the establishment of appropriate limits, encouragement and acknowledgement of the children's accomplishments, cooperating for the children to be able to empathize, through affect, safety and bonding. The need for stable and supportive communities and cultural continuity is linked to the concept that community and culture are foundations for the development of children and their family, considering the care, educational and health aspects in their social network, for the children to gain the feeling of belonging to the family and community^(^ 10 ^)^. The set of these needs entails relevant implications for the promotion of the child's health and physical and emotional safety.

This research was undertaken in Ribeirão Preto-SP-Brazil, in the coverage area of a family health service that belongs to the public primary care network, which attends to a population of about 3800 predominantly young people, whose families live in precious conditions, including immigrant families. 

For the data collection, meetings were held with the professionals from the health services, during which it was verified that there were 27 families in the coverage area with children of less than one year coming from immigrant families. On these occasions, it could be verified that the mothers' life stories were marked by a lot of adversity. In general, their education level was low, they lived in slum regions and came from interior cities in the following Brazilian states: Bahia, Goiás, Maranhão, Alagoas, Pernambuco, Pará and Piauí. The inclusion criteria were: mothers of children under one year registered and monitored at the selected family health service, coming from different Brazilian regions and living in slum regions. The exclusion criteria were: mothers with mental health problems, moving from the coverage area of the selected health service and interrupting the child health monitoring during the first year of life. 

Among the 27 families, 11mothers did not participate in this research (seven were not located after three attempted home visits and four moved from the coverage area of the family health service). The 16 mothers included in the study were between 17 and 29 years of age, with less than eight years of education, and had lived in the city between two months and 15 years. The data collection was based on interviews, which were recorded and held at the participants' homes in 2012. The interviews departed from the following guiding questions: In daily life, what has the care for your son/daughter been like? In your opinion, what are his/her health needs?

The data analysis, based on thematic content analysis, involved the pre-analysis (reading of empirical material to map the reports and meanings the subjects attributed); analysis of the meanings (identification of senses and meanings); elaboration of themes (synthesis of empirical material) and final analysis (discussion of themes)^(^ 11 ^)^. For analytic purposes, thematic units were constructed to apprehend the maternal experiences in care for the child and his/her safety and to identify aspects of the essential needs, highlighting that they do not appear in isolation, but are interwoven and indicate the presence of multiple dimensions for health care.

Approval for the research was obtained from the Research Ethics Committee, in compliance with the recommendations for research involving human beings, with explicit acceptance of the participants through the Free and Informed Consent Form, opinion 439/CEP/CSE-FMRP-USP.

## Results

The results were grouped in thematic units that translate the main care the interviewees reported, linked to the promotion of their children's safety: Ongoing nurturing relationships; Physical protection and safety; Experiences appropriate to child development; Limit setting; Stable and supportive communities.

### Ongoing nurturing relationships

The participants discussed what they consider important for the child to grow and develop in the first year of life, highlighting the relevance of the mother and father's loving presence and engagement in daily care for the child.

*Having the monitoring of the mother and father around. I think that is very important, the mother and father together. *(E2)

*What's important is to be patient, thoughtful, very kind and loving. *(E6)

*I think it's the education I give. It depends on me for them to grow up as good people, on my part and his father's part. *(E5)

The presence of the caregiver and people from the family context is considered to promote the child's safety, as they are trustworthy with a view to an effective care relation.

*I am very concerned with whom I am going to leave them with *[the children]*. When my mother *[maternal grandmother]* is unavailable I don't leave them with anyone. I am afraid of hitting, mistreating, pinching, jumping meal times, those things.* (E6)

*It was not that hard to know how to take care of him, because my mother was nearby, I had the family, I was near his mother *[paternal grandmother]* who helped me a lot, it was no problem. *(E7)

Physical and psychological aspect of child safety at home can be visualized and suggest that the mothers and children need proximity. There are concerns with childcare in order to guarantee favorable conditions to maintain their physical and emotional integrity, in search of nurturing relationships.

### Physical protection and safety

The reports contain aspects related to hygiene, food, disease and accident prevention, which the mothers consider important in daily childcare to prevent diseases.

*Although my house is messy, I take great care with their [children] hygiene. I keep watching everything so he doesn't put it in his mouth, so as not to forget about bathing times. For now, I avoid contact with animals, because he likes ducks and dogs a lot. So you have to keep a close eye. I wash his hands a lot. I try not to let him get barefoot. My mother says that, during the first days, you shouldn't get into the cold, avoid letting him near many people. Sometimes, some flue, something passes easily.* (E6)

*I avoid going out at night, in the dew. The things older people say, you know? 'Don't do this, don't do that'. I even take care of my food? I take care of myself. Because the people say: look, that will give the baby cramps, then I don't eat it. I am trying to eat the healthiest possible things. The vaccines he is taking also protect.* (E3)

*I take good care, don't leave him barefoot, without clothes. I don't give cold baths so as not to catch a cold. *(E11)

The reports also expressed care through concrete actions in terms of domestic accident prevention, such as: avoiding falls, choking and intake of substances or objects, so as to guarantee the child's physical safety and watch out for signs of health problems.

*I always take care of him, I don't leave him near the bedside, I am always taking care of him. I check whether there's some spot on his body, what it is like, you know? *(E1)

*When she slept, I always put her on her side so she wouldn't choke. I took great care to give a bath, so as not to drown or slide. I avoided leaving things she could drink or eat, you know? I put them in a higher place. *(E5)

*Always keeping a close eye on her and never leaving anything small on the floor. Because, as she's crawling, she takes it and can choke on it. I am watching all the time. *(E11)

The care the mothers indicate allows them to promote their children's safety, choosing constant maternal surveillance as a strategy for the early detection of problems at home. 

Besides the care to protect the children, the interviewees highlight the care aimed at preventing mistreatment in childhood, whether due to neglect or physical violence.

*Not taking the child to the doctor, not taking good care, leaving the child completely dowdy at home, not treating the food properly, abandoning the child. That's mistreatment and won't do her any good. *(E14)

*There can't be mistreatment, taking away the childhood. Keep on depriving her *[child]* from playing, depriving from a lot of things, as I see people slapping, what idea does she have of that? You're going to hit her for what? What judgment does she have? I think these things inhibit the child from growing*. (E13)

The mothers indicate actions and some important behaviors for childcare, mentioning forms of interaction that are improbable to promote good emotional development. They also demonstrate caution to offer physical and emotional protection to the child and indicate attitudes that can guarantee the continuity of their safety.

### Experiences appropriate to child development 

The interviewees characterize aspects of the care they deliver as important because they are focused on development, as well as other experiences of the children, which stimulate and add new interactions and learning in the child's daily life. 

*Since birth everything you do is to develop the child. If you talk in the cradle it is to develop, if you put her on your lap when you are breastfeeding and talking, all that is for the child's development.* (E13)

*I noticed that, after she*[child]* started in kindergarten she got smarter. It seems she develops faster, she's talking more. *(E16)

They also indicate the importance of having access to the appropriate forms of stimulation for the child, according to the phase of his/her development.

The interviewed women reported aspects regarding the children's protection, mentioning the aspects that guarantee their rights.

*The child has the right to play, to have fun. *(E11)

*She [child] has to learn what is right and wrong since a very young age, just by talking and teaching. *(E13)

Dialogue and play appear as important tools to maintain the care environment healthy and to give opportunity to the children's singularities. The maternal narratives signal the daily care that cooperate to gain the child's trust, related to gaining self-confidence and to how to feel accepted, heard and cared for. 

### Limit setting

That is one of the areas of need in which conflicts or controversies appear. The participants indicate the importance of setting limits and choices, checking in daily care what contributes to the child's appropriate development. But, at the same time, there are difficulties to set limits, with moments of overprotection, as well as to accept some choices of the child, leading to the imposition of certain care, justified by the understanding that the mother knows what is best for the child.

*In my way of thinking, I think she already has the right to choose what she wants. If she does not want to eat what I oblige her, when it's something good or when it tastes bad, I oblige her to eat. For me it's good, but for her, she thinks it isn't, but I oblige her. Now, like, when she wants to sleep I respect her space, I put her to sleep.* (E5)

*She sleeps with me. So, I think that, even if she sleeps at night in her bed, I won't be able to sleep, because I'll be watching. Even more because it's cold.* (E3)

One aspect that should be highlighted is that children under one year of age, when they sleep with adults, can be exposed to vulnerable situations.

### Stable and supportive communities

The reports indicate the relevance of a support network with professionals from the public Health, Education and Social Service sectors, which help with childcare.

*I think it's easy to take care because I already left the hospital with all information. They taught me a lot.* (E5)

*I do everything the doctors recommend. Now she*[child]*has bronchiolitis. So you need to take care all the time. *(E10)

*Whenever I have some doubt or something happens and my child does not have an appointment, I call the unit and talk to the nurse, I can solve my doubts, my uncertainties. *(E15)

A strong trait of support that was mentioned derives from the health services, with timely access to qualified information for health maintenance.

Reference was made to the need for support for maternal development, with a view to the best childcare.

*If I have education, I'll be able to transmit something to my daughter and she'll be able to complement it in school, it will get easier for her. So I think she'll be able to learn much more. *(E3)

*It seems that, because we study, we get more information. And, when we don't, we don't have any information. So, you take good care, but not as well as who studies.* (E5)

The reports suggest that the support networks, considering education, health, family structure and work, constitute frameworks for the growth and development of the children and their families, in order to seek healthy and trustworthy conditions. At times of uncertainty, the health professionals give directions on the care offered and positive references for the mothers.

In the narratives, there are no noteworthy specificities related to the living conditions. The mothers reported care concerning the safety of all children, independently of their social situation, showing themselves generally capable of providing that care. When referring to their low education level, however, they indicated a feeling of inability to attend to the child's needs more completely, which they aim to compensate for with the support of the health services.

## Discussion

This study shows the importance of acknowledging the parents' participation and responsibility in childcare. In line with other studies^(^ 1 ^-^ 2 ^)^, the narratives are coherent with the idea that the parents, especially the mothers, represent reference figures in childhood, with interaction as the base for the children to build their identity and safety. Because of the dependence inherent in the early stages of the lifecycle, the family has a structural role in attending to the children's needs, such as: food, warmth, shelter, protection and an environment in which they can develop their physical, mental and social skills to a maximum^(^ 1 ^)^.

In daily care at home, according to the maternal perspectives, measures that prevent diseases and other health problems should be prioritized, always keeping an adult alert. Thus, the need for small children to be supervised by careful adults is acknowledged, who should make insightful choices of material and equipment and promote proper modifications in the environment with a view to child safety^(^ 2 ^)^. In fact, many non-intentional (accidents) and intentional (violence) physical injuries happen at home, which implies the need to hold the caregivers accountable for their prevention in that context^(^ 3 ^)^.

The mothers' acknowledgement of the dependence on this permanent supervisory care can be considered appropriate given the children's age range in this study. Nevertheless, the idea is perceived that small children are completely incapable of protecting themselves, culminating in maternal behavior of putting the child to sleep with her for example. This reveals an understanding of child development that can be improved with a view to the promotion of relations that gradually favor the child's autonomy and guarantee his/her safety. The family needs to be prepared to acknowledge the child's development phases and demands, helping to reduce and cope with frustrations.

In the maternal narratives, concerns with acts of violence against the children are observed, showing some vision to deal with the social conditions. Poverty is considered one cause of violence against children^(^ 12 ^)^, resulting from a combination of personal, family, social, economic, political and cultural factors. In the reports, the mothers indicate that they are doing their best within their possibilities, with daily care for the child according to their values and worldviews. That is a positive aspect, as the parents and relatives' good relationship with the children represents a protection factor against violence, besides protection in the development of the child's potentials^(^ 1 ^)^. The results support that other factors, besides financial and social factors, can be related to the child's safety at home. Hence, even in adverse situations, violence against children may not be committed, suggesting that other strategies can be developed to overcome the social conditions. 

It should be highlighted that effective communication and interaction with the children promote their appropriate growth and development. Therefore, an upbringing that guarantees emotional safety is fundamental. Safe environments during the first years of life are related to interactions with key adults and caregivers, as well as with appropriate opportunities for growth, development and learning^(^ 2 ^,^ 6 ^)^. Human brain development is influenced by the environment and by the relations established in early childhood, and the facilitating caregiver-child interactions include emotionally positive attitudes, sensitivity, responsiveness and non-use of physical punishments^(^ 5 ^)^. 

In the promotion of healthy development, at different times in their life, children gain certain skills and experiences. Therefore, the parents' understanding is important, as rushing children in a certain skill or stage of life can often make them slower^(^ 10 ^)^. In addition, the parents should know that individual differences are part of the children's development and that the care should be adapted to these differences^(^ 10 ^)^.

In the childcare process, it is important to know the aspects linked to support and the social network, with people and institutions the parents seek in daily life and in their historical context. The mothers highlight the children's insertion in kindergarten as a support for their growth and development. The mothers sought interactions to help with childcare, seeking health services for occasional care, schedules appointments or to solve doubts. At those moments, the health professionals become references for them, highlighting the role of Nursing. Similarly, Nursing is responsible for transforming these contacts into opportunities to fully analyze the children and their families^(^ 7 ^)^. Child health monitoring that stimulates bonding among child, family and service is vital to prevent problems and promote health, besides creating the possibility for expanded and shared care between family and health services^(^ 7 ^)^.

The social networks are considered articulated sets of relations between the subjects and institutions, and what is expected is that they get consolidated in long-lasting interactions^(^ 13 ^-^ 14 ^)^. Special attention should be paid to the predominant communication standards in the different communities, in which social and community standards and values can mold the mother-child interactions^(^ 15 ^)^. In this study, the maternal narratives show that the construction of strong bonds with the families offers support, but it is important that this is real and truly part of their social network. The construction of long-lasting bonds can be achieved through supportive interactions, following courses the professionals can and should take to encourage and expand the childcare opportunities in their growth and development process. Thus, a strengthened network promotes supportive interactions for the family in childcare^(^ 10 ^)^. Nurses in general guide the parents in making important choices at times of transition, which mold subsequent trajectories in their own and their children's life, giving responses to the vulnerabilities and support to the parents to protect the children^(^ 16 ^)^. In that sense, the skills to allocate resources and strategies to the essential health needs^(^ 8 ^,^ 17 ^)^ are fundamental, knowing and evaluating the family needs^(^ 18 ^)^ with a view to the reduction of social inequities^(^ 5 ^,^ 17 ^)^. 

The health professionals are responsible for enhancing the family's position in the interactions, sharing knowledge and granting support to counter social, individual and institutional vulnerabilities.

## Conclusion

In this study, the elements could be apprehended that facilitate child health promotion at home: the presence and engagement of the parents, constant surveillance for physical and emotional protection; experiences to stimulate child development, nurturing networks for child health care; and the elements that inhibit child safety: limited perception of child development characteristics and singularities of the child, overprotection and difficulties to set limits. In addition, the relevance of considering the maternal perspective on daily childcare was reaffirmed, as a strategy to apprehend aspects related to attendance to the needs to promote growth and development, particularly the promotion of child safety at home. 

The children's safety, permeated by the essential needs, contributes to an effective balance in their growth and development and the elements identified here are important for clinical practice in primary health care.

It should be highlighted that child safety is a complex research problem. Expansion to other studies is needed with a view to observing the care and mechanisms through which the interventions can reduce injuries at home, based on the essential and special needs for comprehensive child health care.
